# Increasing Efficiency of Repetitive Electroacupuncture on Purine- and Acid-Induced Pain During a Three-Week Treatment Schedule

**DOI:** 10.3389/fphar.2021.680198

**Published:** 2021-05-10

**Authors:** Jie Li, Ying Zhang, Peter Illes, Yong Tang, Patrizia Rubini

**Affiliations:** ^1^ School of Acupuncture and Tuina, Chengdu University of Traditional Medicine, Chengdu, China; ^2^ International Collaborative Center on Big Science Plan for Purinergic Signalling, Chengdu University of Traditional Medicine, Chengdu, China; ^3^ Rudolf Boehm Institute for Pharmacology and Toxicology, University of Leipzig, Leipzig, Germany

**Keywords:** electroacupuncture, analgesia, subacute pain, P2X3 receptors, P2X7 receptors, ASIC channels 3

## Abstract

Acupuncture (AP) is an important constituent of the therapeutic repertoire of traditional Chinese medicine and has been widely used to alleviate chronic painful conditions all over the world. We studied in rats the efficiency of electroacupuncture (EAP) applied to the Zusanli acupoint (ST36) as an analgesic treatment over a 3-week period of time on purine (α,β-methylene ATP, dibenzoyl-ATP)- and acid (pH 6.0 medium)-induced pain in the rat paw. The two ATP derivatives stimulated P2X3 and P2X7 receptors, respectively, while the slightly acidic medium stimulated the “acid-sensitive ion channel 3” (ASIC3). It was found that the P2X7 receptor and ASIC-mediated pain was counteracted by EAP with greater efficiency at the end than at the beginning of the treatment schedule, while the P2X3 receptor–mediated pain was not. Our findings have important clinical and theoretical consequences, among others, because they are difficult to reconcile with the assumption that AP is primarily due to the release of peripheral and central opioid peptides causing the well-known tolerance to their effects. In consequence, AP is a convenient therapeutic instrument to treat subacute and chronic pain.

## Introduction

Acupuncture (AP) which is one of the disciplines within the therapeutic repertoire of traditional Chinese medicine (TCM) has been more recently widely used to alleviate chronic painful conditions all over the world (Tang et al., 2016; Tang et al., 2019). In AP, metallic needles are inserted into the subdermal tissue at acupoints and manipulated manually or stimulated electrically (electroacupuncture; EAP). Acupoints are certain acupuncture-sensitive points, the stimulation of which changes the flow of the life energy Qi through the 12 meridians believed to regulate health state according to TCM. Although in the clinical praxis, AP is a means of treating low back pain (Berman et al., 2010; Xiang et al., 2020), chronic neck pain (Seo et al., 2017), musculoskeletal pain (Yuan et al., 2016), migraine (Pei et al., 2019), as well as visceral (Lee et al., 2019) and cancer pain (Chiu et al., 2017), the evidence for its efficiency is, in spite of conducting sham-controlled randomized trials and meta-analyzes even deposited in the Cochrane database, far from being unequivocal. The cautious assessment of the results is based on the following considerations (Madsen et al., 2009; Colquhoun and Novella, 2013): 1) large multicenter clinical trials consistently revealed that the true (verum) and sham AP (stimulation at nonspecific acupoints) do not differ in their effectiveness in decreasing pain levels across multiple chronic pain disorders; 2) the existing differences between AP (verum or sham) and non-AP groups in improvement were only minor; 3) it is hard to understand why AP relieves some types of pain while leaving other types of probably similar etiology unaffected; and 4) interestingly, the strongest evidence for a positive outcome of AP treatment is in case of postoperative nausea and vomiting, conditions not related to pain (Tang et al., 2016).

In contrast to clinical investigations, animal experiments can be easily standardized and evaluated by stringent statistical methods including comparison with a sufficient number of control groups. Hence, convincing evidence for the efficiency of AP on painful conditions in laboratory animals was generated and the mode of action of AP was suggested to include, especially, opioid (Wang et al., 2008; Mayor, 2013; Zhang et al., 2014) and purinergic mechanisms (Burnstock, 2009; Tang et al., 2016, Tang et al., 2019). Classic experiments in mice demonstrated already in the seventies of the last century that the administration of the opioid antagonist naloxone blocked the AP-induced analgesia against noxious heat stimulation (Pomeranz and Chiu, 1976). These findings were further elaborated and confirmed in a range of experimental studies (see Discussion). Somewhat later, in the subcutaneous tissue around an acupoint, AP/EAP was shown to release ATP, which was either enzymatically decomposed to adenosine, stimulating analgesic adenosine A1 receptors (A1Rs) (Goldman et al., 2010; Hurt and Zylka, 2012), or on its own right stimulated ATP-sensitive P2X3 and/or P2X7Rs (Tu et al., 2012; Xu et al., 2016; He et al., 2020). Both A1Rs and P2XRs were suggested to be located either at the terminals of sensory nerve fibers in the periphery or along the pain-conducting fiber tracts in the CNS.

In a previous publication, we investigated the types of purine and acid-sensitive receptors participating in EAP applied to rats/mice and characterized them as belonging to the P2X3, P2X7, ASIC3, and TRPV1 classes by the use of pharmacological agonists/antagonists and knockout mice (Zhang et al., 2020). The aim of the present study was much more circumscribed. Because AP is used to treat chronic pain disorders, it is no wonder that one-time sessions of AP lasting for about 30 min are usually repeated twice a week at the beginning and continued once a week later for a minimum of 12 sessions (Berman et al.,2010; Ernst et al., 2011). Unexpectedly, at least according to our best knowledge, it has not been tested hitherto whether with this therapeutic regimen the efficiency of AP changes in animal experiments. We investigated whether purinergically induced pain (P2X3R and P2X7R stimulation) or mild acidic pain [pH 6.0 medium; stimulation of “acid-sensitive ion channels” (ASICs)] into the rat paw could be alleviated by repetitive EAP over a 3-week period with constant efficiency. We report that the effectiveness of EAP over this period of time gradually increased in case of the P2X7R-mediated and acidic pain, but not in case of the P2X3R-mediated pain. In addition, it was shown that a competitive antagonist at P2X7Rs prevented its activation in a similar manner as EAP did.

## Materials and Methods

### Animals

All animal care and experimental procedures complied with the National Institute of Health Guidelines for the Care and Use of Laboratory Animals and were approved by the Animal Ethics Committee of Chengdu University of Traditional Chinese Medicine. The experiments were performed on adult male Sprague–Dawley rats (180–220 g of weight; 7–8 weeks of age); they experienced at least 1-week acclimation to our laboratory animal observation room in a natural light/dark cycle at 22–24°C with free access to water and food.

### Drug Application Protocols

Phosphate-buffered saline (PBS; Solarbio, Beijing) was prepared by adding 0.1 N HCl or in addition 0.1 N NaOH, if required. PBS at pH 7.4 or 6.0 alone and α,β-methylene ATP (α, β-meATP; 200 nM) or dibenzoyl-ATP (BzATP; 20, 50, and 100 nM; each from Sigma-Aldrich, Saint Louis, MO, United states) dissolved in PBS at pH 7.4, were injected by the intra-plantar (i.pl.) route into the left hind paw of rats in volumes of 100 μl. PBS with or without its algesic constituents was applied immediately after the 3rd measurement of the baseline (−30 min) “paw withdrawal latency” (PWL). In previous experiments, we showed that α,β-meATP (100, 200, and 400 nM) dose-dependently shortened the PWL, with a just maximal effect at 200 nM (Zhang et al., 2020).

### Electroacupuncture

**FIGURE 1:**
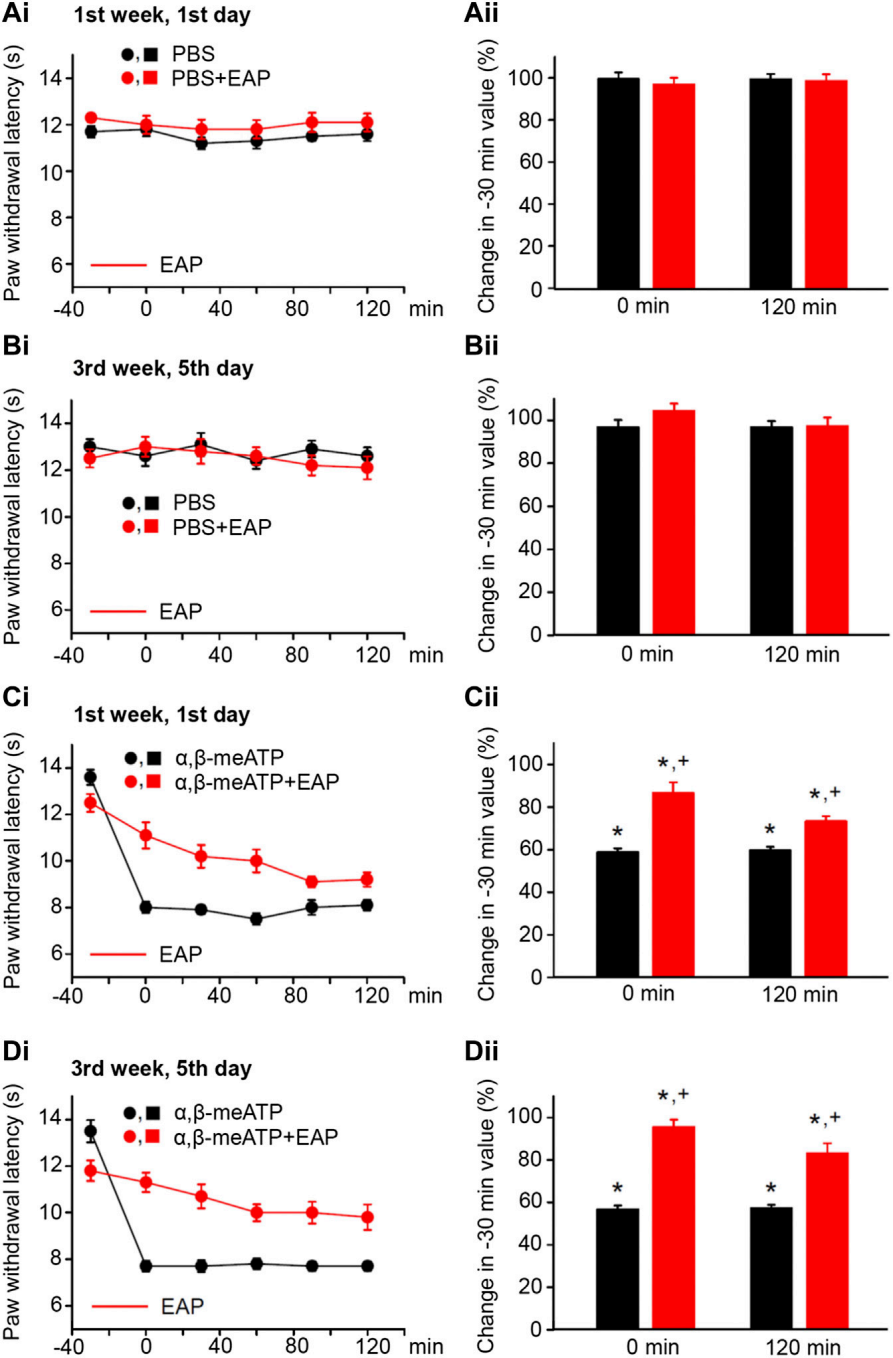
No effect of solvent (PBS, pH 7.4) injection into the left hind paw of rats alone or together with EAP applied to the Zusanli acupoint (ST36) on the “paw withdrawal latency” (PWL); modulation of acute thermal hypersensitivity caused by the injection of α,β-meATP instead of the solvent and its prevention by EAP. The baseline PWL was determined, starting at the −30 min time point, 3 times with 5 min intervals. Solvent or α,β-meATP (200 nM) were injected at −20 min; EAP was delivered from −30 min onwards for 30 min ipsilaterally to the site of injection. Randomly chosen 12 rats were used for each treatment schedule (each symbol or column shows in this and all following figures mean ± SEM of *n* = 12 animals). These rats received solvent/drug injection alone or together with EAP treatment 3 times a week for a total of 9 sessions. PBS and PBS plus EAP do not alter the time–effect relationship of PWL either on the first **(Ai)** or the last application session **(Bi)**. The change of the PWL expressed as a percentage of the baseline PWL (measured at −30 min) as a function of PBS or PBS and EAP at the 0 **(Aii)** and 120 min **(Bii)** time points. α, β-meATP decreases the PWL at 0 and 120 min, both at the first **(Ci)** and the last application sessions **(Di)**. EAP strongly reverses the percentage depression of the EAP at 0 min and less pronouncedly at 120 min irrespective of whether the first **(Cii)** or last application session **(Dii)** is considered. *p < 0.05; statistically significant difference from 100% (the Welch’s test). ^+^ *p* < 0.05; statistically significant difference from the first column in a pair of columns (the Student’s *t* test).

EAP was administered at roughly the same time of the day (10:00 a.m. to 12:00 a.m.) to awake animals, immobilized by two Velcro brand hooks and loop fasteners as well as additional tapes fixed to a wooden block for the duration of EAP only (Zhang et al., 2020). Although immobilization is certainly a stressful stimulus, sham acupuncture applied to the same acupoint but without electrical stimulation (only positioning the needle) or EAP delivered to a non-acupoint, both did not modify the baseline PWL in comparison with the control value measured in previous experiments (compare Figures 1E,H with, e.g., Figures 1A–D of Zhang et al., 2020).

EAP was delivered to the Zusanli acupoint (ST36; located at the left knee, about 6 mm below the fibular head) ipsilateral to the painful stimulus applied to the left hind paw (see above). An electrical current of 1 mA and a frequency of 15 Hz was applied for 30 min, by an “acupoint nerve electrostimulator” (HANS-200, Nanjing Jisheng Medical Technology Co., Jiangsu, China). EAP was delivered through stainless steel needles (2.5 cm long, 0.25 mm diameter; Hwato-Med. Co., Jiangsu, China) introduced 5–8 mm deep below the skin. While one of the electrodes was inserted to the Zusanli acupoint, the other electrode was inserted to a non-acupoint at the basis of the rat’s tail. We strived a high selectivity of stimulation at ST36 and, therefore, have chosen for the second needle a non-acupoint (Zhao et al., 2006; Ye et al., 2020) rather than a neighboring acupoint. Although this results in a larger inter-electrode distance, a field-effect is extremely unlikely in view of the low stimulation current of 1 mA applied. EAP was delivered for 30 min from the 1st measurement of the baseline PWL onwards.

The number of animals in each experimental group equaled 12. This group of 12 rats was randomly divided into two subgroups. Measurements were made on Monday of the first 6 rats and on Tuesday of the second 6 rats; the data obtained were thereafter pooled. This schedule was preserved by determining PWL on the first group of rats always on Monday, Wednesday, and Friday and on the second group of rats on Tuesday, Thursday, and Saturday in total for a duration of 3 weeks. Thus, the painful acidic or purinergic treatment with combined EAP was made 3 times a week. Rats were used only once for the 3-week acupuncture procedure experiment.

### Measurement of Thermal PWL

Responses to thermal laser stimulation were determined at the plantar surface of the left hind paw by using a thermal stimuli instrumentt (PL-200, Techman Software Co., Chengdu, China). The intensity of the light beam was adjusted to 30% and the cut-off time was set to 20 s. All measurements were made in an air-conditioned room (22–24°C). Withdrawal, shaking, or licking of the hind paw was considered as responses to thermal stimulation. Animals were allowed to get accustomed to a transparent plastic enclosure (210 mm × 210 mm × 160 mm) for 30–40 min for three consecutive days each, before the beginning of the test. The baseline PWL (−30 min time point) was determined three times, with 5-min intervals. Then, compounds/drugs were applied i.pl. and the PWL of rats and mice was determined again with the above procedure, every 30 min, including the following six time points: 0, 30, 60, 90, and 120 min. The PWL values were at every time-point the mean of the three determinations.

### Data Analysis

All data were expressed as mean ± SEM of *n* observations, where *n* means the number of animals per group. SigmaPlot 13.0 was used for statistical evaluation. We tested for and found that, when using parametric tests, all sampled distributions satisfied the normality and equal variances criteria. Multiple comparisons between data were performed in case of their normal distribution by one-way ANOVA followed by the Holm–Sidak test. Two data sets were compared by the parametric Student’s *t* test or the non-parametric Welch’s test, as appropriate. A probability level of 0.05 or less was considered to be statistically significant.

## Results

### Repetitive Electroacupuncture and α,β-Methylene ATP-Induced Pain

In the first series of experiments, we tested whether during standard experimental conditions, PBS at pH 7.4 (control), injected into the left hind paw of rats, changed the PWL from baseline (−30-min time-point) over the 120 min observation period. The first (1st week, 1st day; Figure 1Ai) and the last (3rd week, 5th day; Figure 1Bi) measurements of the PWL documented no apparent changes from the baseline value. Similarly, EAP applied for 30 min did not appear to influence the baseline PWL. Because in these and all further measurements at different days the baseline PWL showed rather pronounced variability, we calculated the percentage change of the PWL at the 0 and 120-min time points from baseline. In fact, neither PBS nor PBS plus EAP caused any statistically significant alteration either at 0 min or at 120 min (Figures 1Aii,Bii).

According to our expectations, the P2X1 and P2X3R agonist α,β-meATP caused pain, when injected into the left hind paw of rats; this was due to the stimulation of P2X3Rs localized at the terminals of subcutaneous sensory C-fibers (Chizh and Illes, 2001; Wirkner et al., 2007). Our previous experiments confirmed by use of the highly selective P2X3 and P2X7R antagonists A317491 and A438079, respectively, that under our experimental conditions α,β-meATP activates only the former receptor-type (Zhang et al., 2020). Hence, α,β-meATP (200 nM) caused a marked decrease of PWL from the baseline to its 0-min value and this decrease was stable over the subsequent 120 min, irrespective of whether measurements were at the beginning or at the end of the 3-week treatment schedule (Figures 1Ci,Di; Figures 1Cii,Dii, first column of each pair of columns). EAP depressed the pain caused by α,β-meATP at 0 min, both at the beginning and at the end of the 3-week treatment regimen (Figures 1Cii,Dii, second column of each pair of columns). The effect of EAP showed a tendency to increase when it was tested against the α,β-meATP–induced pain at the 120-min time point, from the 1st week, 1st day (depression to 73.3 ± 2.5%) to the 3rd week, 5th day (depression to 83.2 ± 4.7; *n* = 8; *p* > 0.05; Student’s *t* test) of the treatment procedure, although it did not reach the level of statistical significance. In conclusion, EAP was active in preventing the decrease of PWL by α,β-meATP during a single treatment session, but this effect did not change when EAP was applied repetitively for 3 weeks.

### Repetitive Electroacupuncture and Dibenzoyl ATP–Induced Pain

**FIGURE 2:**
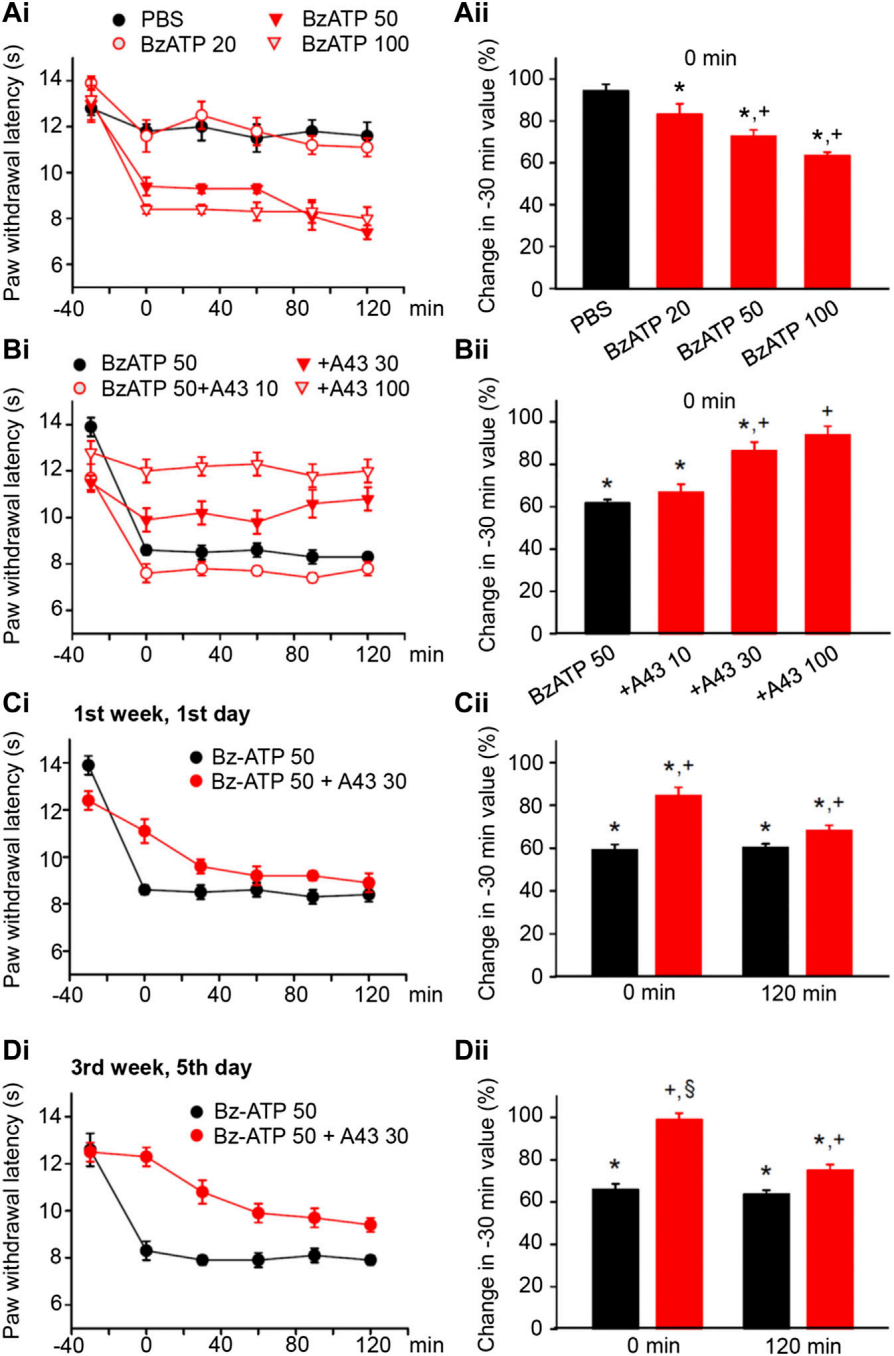
Modulation of acute thermal hypersensitivity (measured as the duration of the PWL) caused by the injection of BzATP into the left hind paw of rats; its prevention by a competitive antagonist at P2X7Rs applied to the same site as the agonist. The baseline PWL was determined, starting at the −30 min time point, 3 times with 5 min intervals. Solvent or BzATP (20, 50, and 100 nM) alone, or in the case of BzATP (50 nM) together with A438079 (10, 30, or 100 nM) were injected at −20 min. Randomly chosen 12 rats were used for each treatment schedule shown here. No effect of solvent (PBS, pH 7.4) injection but a dose-dependent shortening of the PWL by BzATP (20, 50, and 100 nM). **(A)** Time–effect relationships of PWL **(Ai)** and the change of PWL expressed as a percentage of the baseline PWL (measured at −30 min) at the 0 min time point **(Aii)**. Dose-dependent prevention of the BzATP (50 nM) effect by coapplied A438079 (10, 30, and 100 nM). **(B)** Time–effect relationships of PWL **(Bi)** and the change of PWL expressed as a percentage of the baseline PWL (measured at −30 min) at the 0-min time point **(Bii)**. In the following experiments, the rats received solvent/drug injection 3 times a week for a total of 9 sessions. The prevention by A438079 (30 nM) of the BzATP (50 nM) induced decreases of the PWL at 0 and 120 min, both at the first **(Ci)** and the last application sessions **(Di)** in the course of 3 weeks. A438079 strongly reverses the percentage depression of the EAP at 0 min and less pronouncedly at 120 min. However, the effect of A438079 increases at the 0 -min time point from the first to the last application session. *p < 0.05; statistically significant difference from 100% (Welch’s test). ^+^ *p* < 0.05; statistically significant difference from the first column in a pair of columns (Student’s *t* test). §*p* < 0.05; statistically significant difference from the corresponding effect of BzATP plus A438079 in **(Cii)** (Student’s *t* test).

BzATP, the prototypic agonist of P2X7Rs also produced pain, when injected into the left hind paw of rats (Bhattacharya et al., 2011). BzATP stimulates P2X1 and P2X3Rs with higher efficiency than P2X7Rs, but on the other hand, it is a stronger agonist than ATP itself at P2X7Rs (Jarvis and Khakh, 2009; Illes et al., 2021). BzATP (20, 50, and 100 nM) dose-dependently shortened the PWL at the 0-min time point, when compared with the baseline value determined at the −30-min time point (Figures 2Ai,ii). From −30 min onwards, the effect of BzATP at all doses tested remained rather stable over the following 60 or even 120 min (Figure 2Ai). Subsequently, we selected a dose of BzATP (50 nM) which evoked a submaximal decrease of PWL and combined it with increasing doses (10, 30, and 100 nM) of the competitive P2X7R antagonist A438079 (Figures 2Bi,ii). This manipulation caused less shortening of the PWL, in clear dependence on the gradually increasing doses of A438079, unequivocally confirming that BzATP acts as an algesic agent through the stimulation of P2X7Rs.

Then, we applied BzATP (50 nM) with the same protocol as previously α,β-meATP (200 nM) and noticed that P2X7R activation caused during a 3-week period of treatment both at the 0 and 120-min time points a reproducible decrease in the PWL (Figures 2Ci,Di; for statistical evaluation see first columns in each pair of columns in Figures 2Cii,Dii). Interestingly the antagonistic effect of A438079 (30 nM) clearly increased from the beginning till the end of the 3-week treatment regimen only at the 0 -min time point (Figures 2Ci,Di; for statistical evaluation, see second columns in each pair of columns in Figures 2Cii,Dii).

**FIGURE 3:**
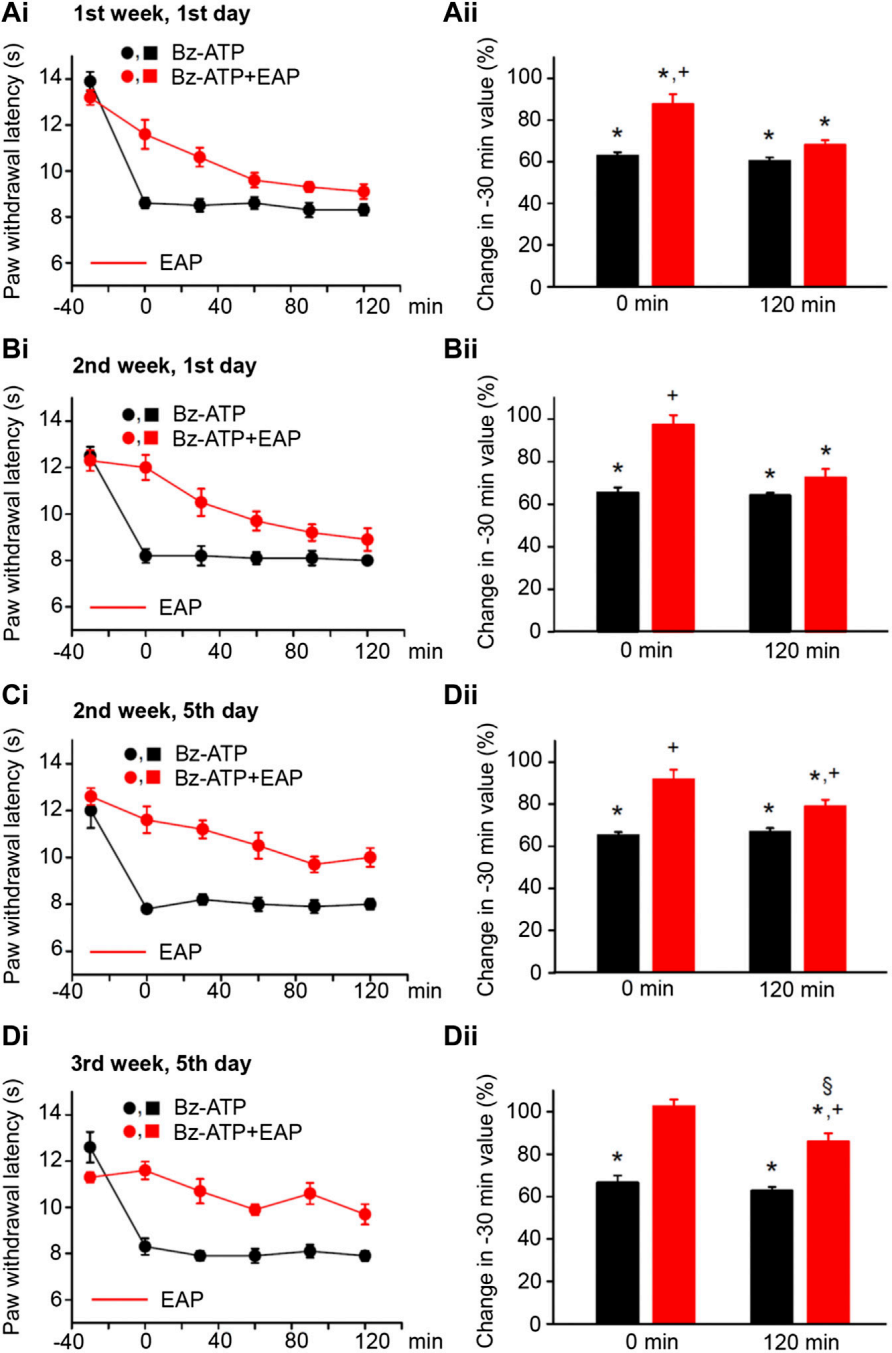
Modulation of acute thermal hypersensitivity caused by the injection of BzATP into the left hind paw of rats and its prevention by EAP applied to the ipsilateral Zusanli acupoint (ST36) on the PWL. Each group of 12 rats received BzATP (50 nM) injection or in addition EAP treatment for 30 min 3 times a week for a total of 9 sessions. All other experimental details were identical with those described in the legend to Figure 1. The change in the time–effect relationship of the PWL on selected application sessions; 1st week, 1st day **(Ai)**; 2nd week, 1st day **(Bi)**; 2nd week, 5^th^ day **(Ci)**; and 3rd week, 5th day **(Di)**. The change of the PWL was expressed as a percentage of the baseline PWL (measured at −30 min) depending on the application of BzATP or BzATP plus EAP at the 0 and 120 min time points **(Aii–Dii)**. BzATP decreases the PWL at all application sessions both at 0 min and 120 min. EAP strongly reverses the percentage depression by BzATP at 0 min and less pronouncedly at 120 min; the extent of this depression inversely correlates with the increasing number of sessions. *p < 0.05; statistically significant difference from 100% (Student’s *t* test). ^+^ *p* < 0.05; statistically significant difference from the first column in a pair of columns (Welch’s test). §*p* < 0.05; statistically significant difference from the corresponding effect of BzATP plus EAP in **(Aii)** (one-way ANOVA followed by the Holm–Sidak test; F = 5.301).

By contrast, EAP depressed the effect of BzATP at 0 min and caused a gradually increasing analgesia at 120 min, when the treatment sessions were repeated 9 times in total, 3 times every week. At the beginning (1st week, 1st day) EAP depressed the effect of BzATP to 68.0 ± 2.4% of baseline, while at the end (3rd week, 5th day) the depression was only to 85.9 ± 3.9% (*n* = 12 each; *p* < 0.05; One-way ANOVA, the Holm–Sidak test). With BzATP we show the 1st (Figures 3Ai,ii), 4th (Figures 3Bi,ii), 5th (Figures 3Ci,ii), and last (Figures 3Di,ii) measurements during the treatment schedule, which document that the 120-min values with EAP were to 68.0 ± 2.4%, 72.5 ± 4.0%, 79.0 ± 3.0%, and 85.9 ± 3.9% of baseline (*n* = 12 each).

### Repetitive Electroacupuncture and Acidification-Induced Pain

The pain-sensing C-fiber terminals are also endowed with the TRPV1 member of the transient receptor potential (TRP) protein superfamily (Jardin et al., 2017; Moran and Szalasi, 2018) as well as the acid-sensing ion channel 1a (ASIC1a) and ASIC3 members of the ASIC family (Wemmie et al., 2013; Cheng et al., 2018). Our previous experiments suggested that low-threshold acidic pain (∼pH 6.0) was mediated by ASIC3/TRPV1 channels while high-threshold acidic pain (∼pH 4.0) was mediated exclusively by TRPV1 channels; only the low-threshold pain was counteracted by EAP (Zhang et al., 2020). In fact, under the present experimental conditions, two selective ASIC3 antagonists, APETx2 and UGR9-1 relieved the pain induced by i.pl. injection of a pH 6.0 PBS, while the TRPV1 antagonist capsazepine also relieved the pain induced by i. pl. injection of pH 4.0 PBS.

**FIGURE 4:**
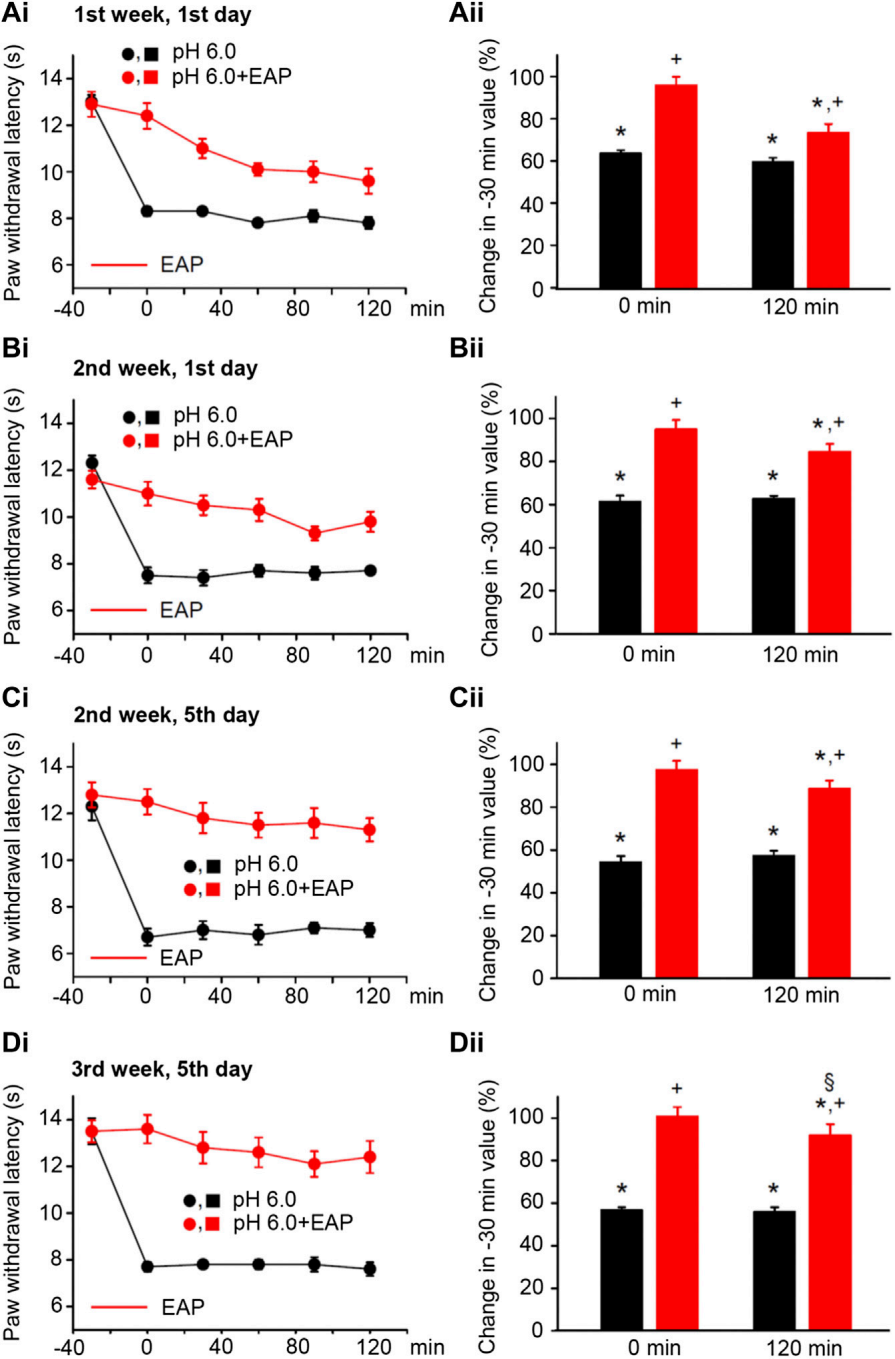
Modulation of acute thermal hypersensitivity caused by the injection of pH 6.0 PBS into the left hind paw of rats and its prevention by EAP applied to the ipsilateral Zusanli acupoint (ST36) on the PWL. Each group of 12 rats received the injection of pH 6.0 PBS, or in addition EAP treatment for 30 min, 3 times a week for a total of 9 sessions. All other experimental details were identical with those described in the legend to Figure 1. The change in the time–effect relationship of PWL on selected application sessions; 1st week, 1st day **(Ai)**; 2nd week, 1st day **(Bi)**; 2nd week, 5th day **(Ci)**; and 3rd week, 5th day **(Di)**. The change of the PWL was expressed as a percentage of the baseline PWL (measured at −30 min) depending on the application of pH 6.0 PBS or pH 6.0 PBS plus EAP at the 0 and 120 min time points **(Aii–Dii)**. The pH 6.0 PBS decreases the PWL at all application sessions both at 0 min and 120 min. EAP strongly reverses the percentage depression by the acidic medium at 0 min and less pronouncedly at 120 min; the extent of this depression inversely correlates with the increasing number of sessions. *p < 0.05; statistically significant difference from 100% (Student’s *t* test). ^+^ *p* < 0.05; statistically significant difference from the first column in a pair of columns (Welch’s test). §*p* < 0.05; statistically significant difference from the corresponding effect of BzATP plus EAP in **(Aii)** (one-way ANOVA followed by the Holm–Sidak test; F = 3.648).

In consequence, we induced pain by injecting pH 6.0 PBS into the left hind paws or rats by following the same experimental protocol as used previously with the other algogenic stimuli. pH 6.0 PBS caused a depression of the PWL comparable to that obtained with BzATP during the 3-week duration treatment regimen both at the 0- and 120-min time points (Figures 4Ai,Di; for statistical evaluation see first columns in each pair of columns in Figures 4Aii,Dii). Again, EAP depressed the effect of the acidic PBS only to 73.4 ± 4.1% of baseline at the beginning, but to 92.0 ± 5.1% of it at the end of the 3-week treatment schedule (*n* = 12 each; *P* < 0.05; One-way ANOVA, the Holm–Sidak test). Acidification to pH 6.0 at 120 min caused gradually lower percentage inhibition at the 1st (Figures 4Ai,ii), 4th (Figures 4Bi,ii), 5th (Figures 4Ci,ii), and last (Figures 4Di,ii) measurements, which document that the 120-min values with EAP were 73.4 ± 7.1%, 84.4 ± 3.7%, 88.6 ± 3.9%, and 92.0 ± 5.1% (*n* = 12 each), respectively.

## Discussion

The main finding of this study is that repetitive application of EAP to the ipsilateral Zusanli acupoint (ST36) for the duration of 3 weeks increased the efficiency of analgesia in case of P2X7R-mediated and acidic pain, but left it grossly unaltered in case of P2X3R-mediated pain. Moreover, EAP appeared to prevent purinergic pain in a similar manner as a competitive antagonist of P2X7Rs did, because in both cases treatment for 3 weeks increased the pain relieving effect. These findings have important clinical consequences because EAP is used as a treatment of chronic pain and in consequence any decrease in its efficiency with time would be a hindrance for therapeutic applicability.

Acute cutaneous pain is accompanied by a rapid drop in tissue pH and injection of acidic saline subcutaneously or intramuscularly results in the development of localized pain both in rodents and human subjects (Ugawa et al., 2002; Karczewski et al., 2010). It has repeatedly been shown that acid-sensing homomeric ASIC channels (ASIC1a and ASIC3; Deval and Lingueglia, 2015; Gründer and Chen, 2010) and TRP channels (TRPA1 and TRPV1; Caterina et al., 1997; Dai, 2016) respond to local acidosis during inflammatory and cancer pain (Deval and Lingueglia, 2015; Dai, 2016). Similarly, the injection of ATP or its release from the intracellular space during all types of tissue damaging conditions causes acute or chronic pain states (Chizh and Illes, 2001; Burnstock, 2009). Homomeric P2X3 and P2X7Rs and heteromeric P2X2/3Rs (North, 2002; Wirkner et al., 2007) mediate inflammatory, neuropathic, visceral, and cancer pain (Burnstock 2013, Burnstock, 2016). Interestingly, ASIC3 and P2X3Rs appear to interact with each other in response to protons and ATP, by forming an ASIC3/P2X3 “cognate” receptor, establishing a tight relationship between acidic and purinergic mechanisms in pain induction (Stephan et al., 2018).

AP raised the levels of opioid peptides in the cerebrospinal fluid of human volunteers (Sjölund et al., 1977). Similarly, in uninjured rats, low frequency EAP released β-endorphin and enkephalins, while high frequency EAP released dynorphins to suppress nociception, as assessed by the tail flick test (Han, 2003). The complete Freund’s adjuvant (CFA)–induced shortening of the PWL in rats was relieved by EAP; this was blocked by µ- but not κ-opioid receptor antagonist injection into the rostral ventrolateral medulla, a brain site which is critical for the supraspinal modulation of dorsal horn nociceptive transmission (Zhang et al., 2011).

In addition to central opioid mechanisms, their peripheral counterparts also appear to participate in EAP analgesia (Zhang et al., 2014; Tang et al., 2016). Intraplantar injection of naloxone or selective antagonists at µ-, δ-, and κ-opioid receptors before EAP dose-dependently blocked the inhibition of mechanical hyperalgesia (Sekido et al., 2003; Taguchi et al., 2010). Consistent with these results, intraplantar naloxone methiodide, a peripherally acting opioid receptor antagonist, eliminated the EAP-induced inhibition of thermal hyperalgesia in a CFA-treated rat (Zhang et al., 2005). It was concluded that EAP released in inflamed tissue from lymphocytes, monocytes/macrophages, and granulocytes opioids which activated their receptors at the terminals of pain-sensing nerve terminals (Cabot et al., 1997; Rittner et al., 2001). As demonstrated by Han et al. (1981), an unavoidable consequence of the opioid receptor mediation of EAP is tolerance to its repetitive application and also cross-tolerance with morphine. However, this phenomenon would create serious difficulties in case of chronic EAP application because of gradual loss of efficiency.

An alternative, more recent hypothesis is based on the AP/EAP–induced release of ATP by the mechanical stimulation by AP needles of cutaneous/subcutaneous mast cells (Cui et al., 2018; Shen et al., 2020). The local outflow of ATP and a whole range of ATP metabolites (ADP/AMP/adenosine) have been measured in the neighboring interstitium by a microdialysis probe after needling of the Zusanli acupoint of mice (Goldman et al., 2010; Takano et al., 2012). It was shown that adenosine, the enzymatic degradation product of ATP stimulated inhibitory A1 adenosine receptors localized at the nerve terminals of sensory afferents (the peripheral terminals of dorsal root ganglion neurons; DRGs) otherwise conveying painful information to the dorsal horn spinal cord. In accordance with this assumption the local application of a slowly degradable synthetic A1R agonist (2-chloro-N^6^-cyclopentyladenosine; CCPA) into the Zusanli acupoint inhibited mechanical and thermal allodynia in the CFA model of inflammatory pain (Goldman et al., 2010; Zylka, 2011). The effect of AP was missing in A1R knockout mice. Further, injection of deoxycoformycin, which inhibits the enzyme AMP deaminase, increased the AP-induced accumulation of adenosine around the Zusanli acupoint and prolonged the antinociceptive effect of AP (Goldman et al., 2010).

ATP released by AP/EAP may cause analgesia both by acting indirectly *via* the release of adenosine, or directly *via* stimulating P2X3 or P2X7Rs in the periphery or along the central pain-conducting pathways (He et al., 2020). EAP has been shown to alleviate pain and downregulate the previously increased P2X3R protein and P2X3R current amplitudes in acutely dissociated DRGs prepared from rats which underwent chronic constriction injury of their sciatic nerves (the neuropathy model; Tu et al., 2012; Wang et al., 2014; Liang et al., 2019; Du et al., 2020) or received CFA injection into their paws (the inflammatory pain model; Xiang et al., 2020). Similarly, the chronic constriction injury– or neck incision–induced pain and the accompanying spinal overexpression of inflammatory cytokines (as typical responses to P2X7R stimulation) as well as increase in P2X7R mRNA were sensitive to EAP (Xu et al., 2016; Gao et al., 2017).

In contrast to the involvement of opioid and purine receptors in EAP-induced analgesia, the evidence for the participation of pH-sensitive receptors (ASICs and TRPs) is quite restricted. The repeated intramuscular injection of acidic medium with a pH of 4.0 caused inflammatory fibromyalgia, beneficially modified by EAP (Chen et al., 2011; Walder et al., 2011). Furthermore, mild acidic pain induced by the injection of a pH 6.0 PBS into the rat paw was prevented by EAP (Zhang et al., 2020).

In perfect accordance with the above literature data, in the present experiments, thermal hypersensitivity mediated by either P2X3Rs, P2X7Rs, or ASIC3 channels was shown to be sensitive to EAP stimulation. Moreover, this sensitivity to EAP markedly increased in case of P2X7R- and ASIC3-mediated pain in the course of a 3-week treatment regimen, while the modulation of P2X3R-mediated pain was much more stable during the same period of time. Our data are difficult to reconcile with the hypothesis that opioid peptides are in the first line responsible for EAP-induced analgesia and favor the clinical applicability of EAP as a therapeutic procedure alleviating subacute/chronic pain.

## Data Availability

The raw data supporting the conclusion of this article will be made available by the authors, without undue reservation.
